# A New Competitor with Homæopathy

**Published:** 1848-07

**Authors:** 


*A new Competitor with Homœopathy. Isopathy,*—A new German humbug has lately appeared upon the tapis, which bids fair to make a strong appeal to the affections of those who are ever greedy for medical delusions. The following account of the would-be-rival of Homœopathy, is taken from the London Medical Gazette:—

" A new medical doctrine has appeared on the horizon, and it is Germany again, *Alma parens rerum*, which enriches the world with this benefit. Homœopathy, magnetism, and phrenology, salute their new sister under the harmonious name of Isopathy. Dr. Hermann is the prophet of this doctrine, which is grounded on the following principle:—Every diseased organ has its remedy in the same organ; thus if you have disease of liver, eat liver; if a headache, eat brain; if you suffer in the bladder or kidneys, nourish yourself on bladder and kidneys; if the testicles be disordered eat testicles. As the organs may not appear very tempting to certain squeamish persons, M. Hermann has made tinctures of them, which his patients take in spoonfuls, under the scientific names of *Stomachine, Cystine, Testiculina, Umbria, &c.* The work published at Augsburg, contains fifty cases of radical cures. Go! young doctrine, increase and prosper—thou willt doubtless be called to high destinies!"

